# Supplementary material for: A Mitochondrial Genome Phylogeny of Cleridae (Coleoptera, Cleroidea)
Source: Insects. 2022 Jan 24;13(2):118. doi: 10.3390/insects13020118 (PMC8878092; doi:10.3390/insects13020118)
Supplement: Supplementary file 1 [file Supplementary Figures.pdf › Supplementary Figures.pdf]

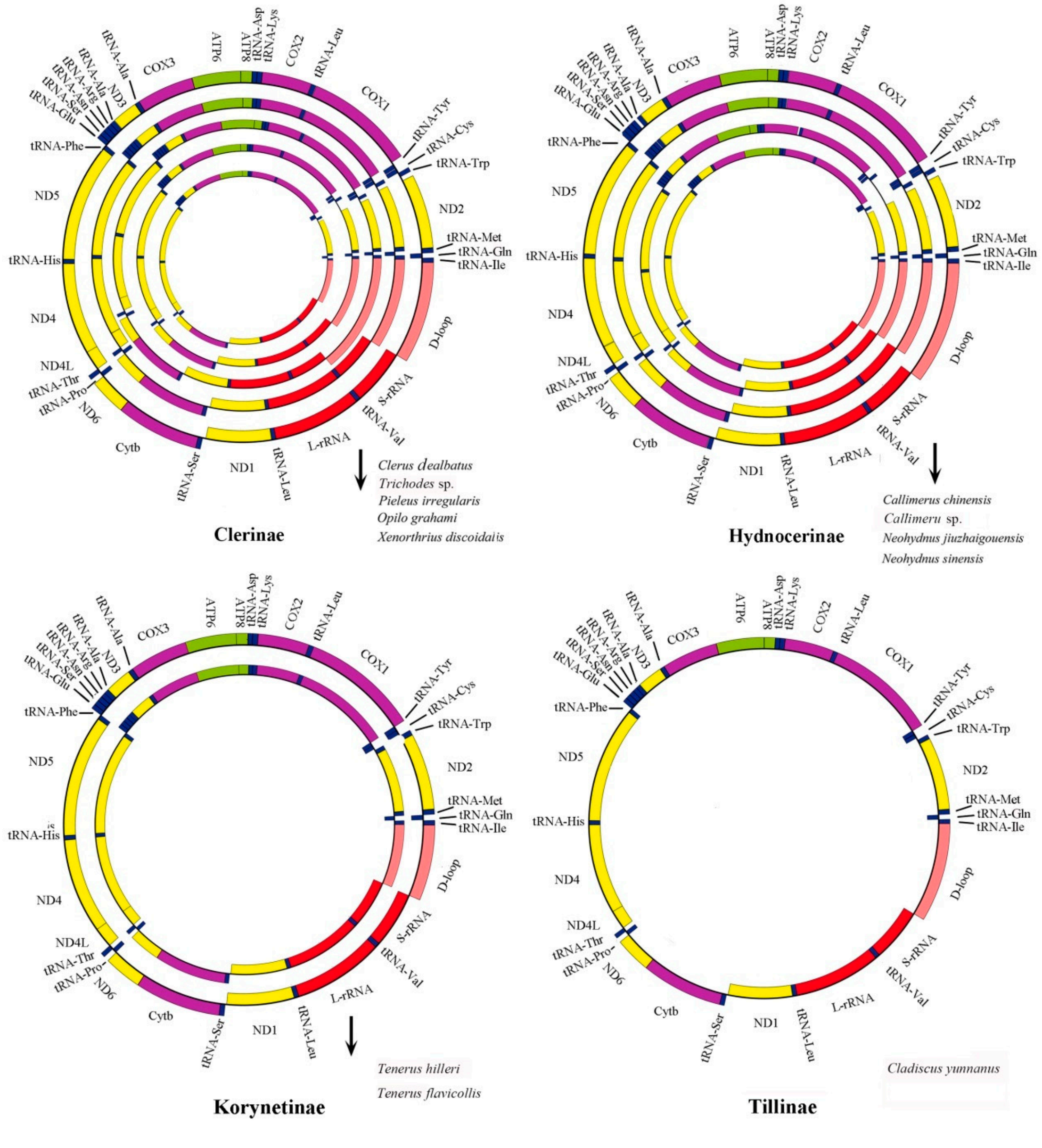

Figure S1.Circular maps of the completemitogenomes of four subfamilies of Cleridae. Different color represents different types of genes and regions. Genes shownat the inner circle are located on the N-strand, while those at the outer circle on the J-strand.

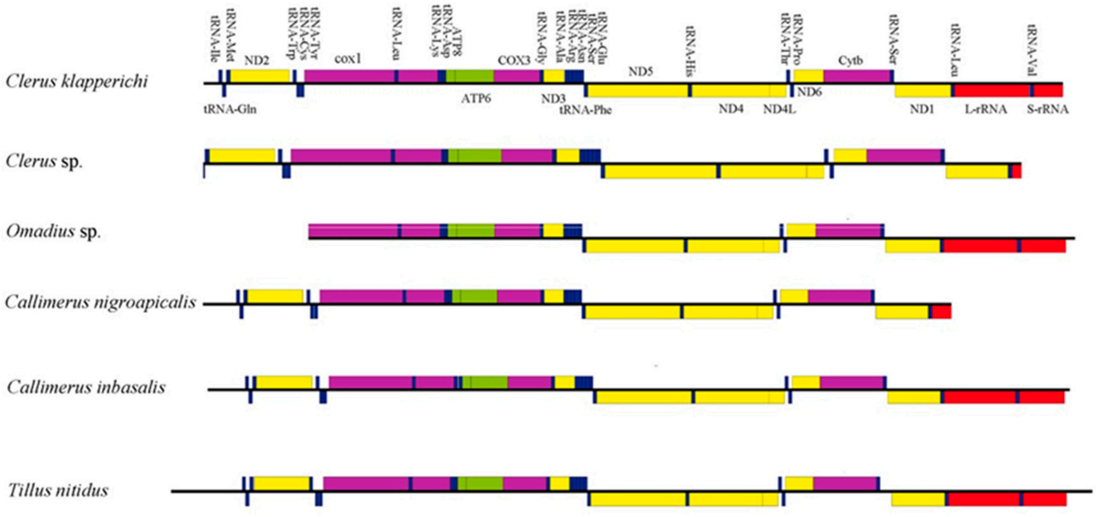

Figure S2. Maps of the nearly complete mitogenomes of four subfamilies of Cleridae. Different color represents different types of genes and regions.

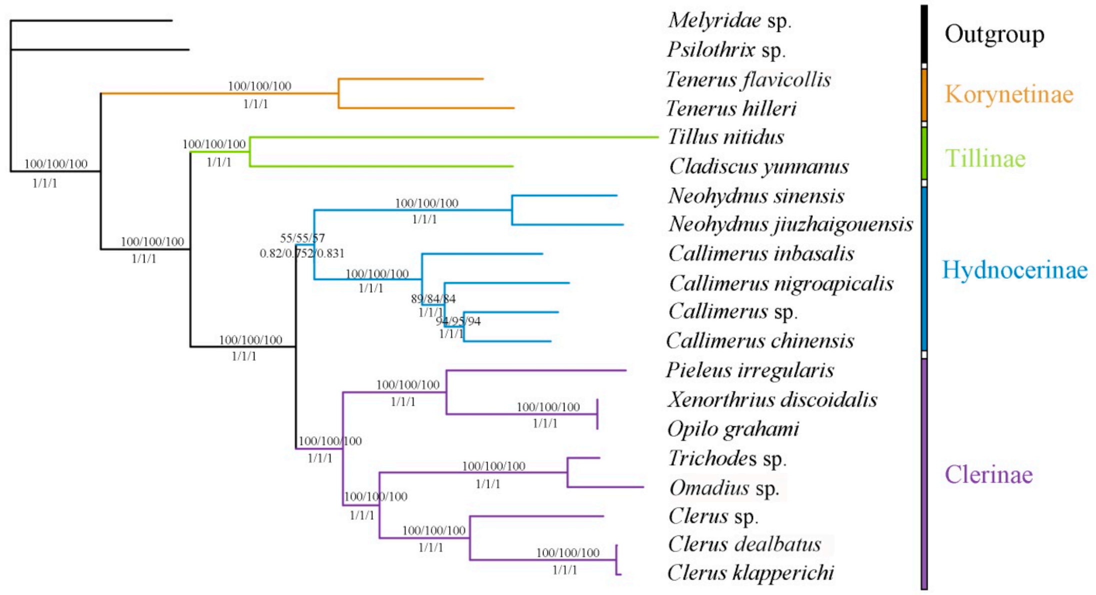

Figure S3. Phylogenetic tree of Cleridae inferred from the BI and ML analyses of the PCGRNA (left), PCGrRNA (middle) and PCG (right) datasets. Numbers at the branches are bootstrap values (upper) or posterior probabilities (lower).
