# Supplementary material for: A Mitochondrial Genome Phylogeny of Cleridae (Coleoptera, Cleroidea)
Source: Insects. 2022 Jan 24;13(2):118. doi: 10.3390/insects13020118 (PMC8878092; doi:10.3390/insects13020118)
Supplement: Supplementary file 1 [file insects-13-00118-s001.zip › Supplementary Tables.pdf]

Table S1: Best models calculated by BI of four mitochondrial datasets

PCGRNAdataset

Partitions	Best Model	Partition names
P1	HKY+G	trnL2,trnC
P2	GTR+I+G	trnS2, trnF,trnY,trnP
P3	GTR+G	trnR,rrnL
P4	GTR+I+G	trnH, rrnS
P5	GTR+I+G	trnE,trnS1,trnT,trnA,trnD, trnG,trnN
P6	GTR+G	trnI,cox2_pos1,
P7	GTR+I+G	atp8_pos2,trnM,trnK
P8	GTR+I+G	trnL2, nad5_pos2
P9	HKY+G	trnQ
P10	F81+I	trnV
P11	HKY+I+G	trnW
P12	GTR+I+G	atp6_pos1, cytb_pos1, cox3_pos1
P13	GTR+I	atp6_pos2,nad1_pos2,
P14	HKY+I+G	cox3_pos3,atp6_pos3,nad3_pos3
P15	HKY+G	atp8_pos1
P16	HKY+I+G	nad2_pos3, atp8_pos3
P17	GTR+I	cox1_pos1
P18	F81+I+G	cox1_pos2
P19	GTR+G	cox1_pos3
P20	GTR+I+G	cox3_pos2,cox2_pos2, cytb_pos2
P21	HKY+I+G	cox2_pos3
P22	HKY+G	cytb_pos3
P23	GTR+I+G	nad1_pos1
P24	HKY+G	nad1_pos3
P25	GTR+I+G	nad6_pos1,nad2_pos1,nad3_pos1
P26	GTR+I+G	nad3_pos2,nad6_pos2,nad2_pos2
P27	GTR+I+G	nad5_pos1,nad4L_pos1,nad4_pos1
P28	GTR+I+G	nad4L_pos2,nad4_pos2,
P29	HKY+G	nad4L_pos3
P30	GTR+G	nad5_pos3,nad4_pos3
P31	HKY+G	nad6_pos3

PCGrRNA dataset

Partitions	Best Model	Partition names
P1	GTR+G	rrnL
P2	GTR+I+G	rrns
P3	GIR+G	cox2_pos1
P4	GTR+I+G	cox3_pos2,cytb_pos2, cox2_pos2
P5	HKY+I+G	cox2_pos3
P6	GTR+I+G	atp6_pos1,cytb_pos1, cox3_pos1
P7	HKY+I+G	nad3_pos3,cox3_pos3, atp6_pos3
P8	HKY+G	cytb_pos3
P9	GTR+I+G	nad1_pos1
P10	GTR+I	nad1_pos2, atp6_pos2
P11	HKY+G	nad1_pos3
P12	GTR+I+G	atp8_pos1,nad3_pos1, nad6_pos1, nad2_pos1
P13	GTR+I+G	nad3_pos2, nad6_pos2, nad2_pos2

P14	HKY+I+G	nad2_pos3, atp8_pos3
P15	GTR+I+G	nad5_pos1, nad4_pos1, nad4L_pos1
P16	GTR+I+G	nad4L_pos2, nad4_pos2
P17	HKY+I+G	nad4L_pos3
P18	GTR+I+G	nad5_pos3, nad4_pos3
P19	GTR+I+G	nad5_pos2
P20	HKY+G	nad6_pos3
P21	GTR+I+G	atp8_pos2
P22	GTR+G	cox1_pos1
P23	GTR	cox1_pos2
P24	GTR+G	cox1_pos3
**PCG dataset**		
Partitions	Best Model	Partition names
P1	GTR+I+G	atp6_pos1,cytb_pos1, cox3_pos1
P2	GTR+I+G	atp6_pos2, nad1_pos2
P3	HKY+I+G	cox2_pos3,nad3_pos3, cox3_pos3, atp6_pos3
P4	HKY+G	atp8_pos1
P5	GTR+G	atp8_pos2
P6	HKY+I+G	nad2_pos3, atp8_pos3
P7	GTR+I	cox1_pos1
P8	F81+I+G	cox1_pos2
P9	GTR+G	cox1_pos3
P10	GTR+G	cox2_pos1
P11	GTR+I+G	cox3_pos2, cox2_pos2, cytb_pos2
P12	GTR+G	cytb_pos3
P13	GTR+I+G	nad1_pos1
P14	HKY+G	nad1_pos3
P15	GTR+I+G	nad6_pos1, nad3_pos1, nad2_pos1
P16	GTR+I+G	nad3_pos2, nad2_pos2, nad6_pos2
P17	GTR+I+G	nad5_pos1, nad4L_pos1, nad4_pos1
P18	GTR+I+G	nad5_pos2, nad4L_pos2, nad4_pos2
P19	HKY+G	nad4L_pos3
P20	GTR+G	nad5_pos3, nad4_pos3
P21	GTR+G	nad6_pos3
**PCG12 dataset**		
Partitions	Best Model	Partition names
P1	GTR+I+G	atp6_pos1,atp8_pos2,cox3_pos1,cob_pos1
P2	GTR+I	atp6_pos2, cox1_pos2
P3	GTR+I+G	nad6_pos1, atp8_pos1,nad2_pos1,nad3_pos1,
P4	GTR+I	cox1_pos1
P5	GTR+G	cox2_pos1
P6	GTR+I+G	cob_pos2, cox2_pos2, cox3_pos2
P7	GTR+I+G	nad1_pos1,nad4_pos1, ad4l_pos1,nad5_pos1,
P8	GTR+I+G	nad1_pos2,nad4_pos2,ad4l_pos2,nad5_pos2,
P9	GTR+I+G	nad6_pos2, nad2_pos2, nad3_pos2

Table S2: Best models calculated by IQ of four mitochondrial datasets

**PCGRNAdataset**		
Partitions	Best Model	Partition names
P1	HKY+F+G4	trnL2,trnC
P2	TIM+F+G4	trnS2, trnF,trnY,trnP
P3	GTR+F+G4	trnR,rrnL
P4	GTR+F+I+G4	trnH, rrnS
P5	TPM2u+F+I+G4	trnE,trnS1,trnT,trnA,trnD, trnG,trnN
P6	TIM3+F+G4	trnI,cox2_pos1,
P7	TPM2+F+G4	atp8_pos2,trnM,trnK
P8	GTR+F+G4	trnL2, nad5_pos2
P9	HKY+F+G4	trnQ
P10	F81+F+I	trnV
P11	TIM3e+G4	trnW
P12	GTR+F+G4	atp6_pos1, cytb_pos1, cox3_pos1
P13	TIM+F+I	atp6_pos2,nad1_pos2,
P14	HKY+F+I+G4	cox3_pos3,atp6_pos3,nad3_pos3
P15	HKY+F+G4	atp8_pos1
P16	HKY+F+I+G4	nad2_pos3, atp8_pos3
P17	TIM2+F+I	cox1_pos1
P18	F81+F+I	cox1_pos2
P19	HKY+F+G4	cox1_pos3
P20	TIM+F+I+G4	cox3_pos2,cox2_pos2, cytb_pos2
P21	HKY+F+I+G4	cox2_pos3
P22	HKY+F+G4	cytb_pos3
P23	TPM2u+F+I+G4	nad1_pos1
P24	HKY+F+G4	nad1_pos3
P25	GTR+F+I+G4	nad6_pos1,nad2_pos1,nad3_pos1
P26	TVM+F+I+G4	nad3_pos2,nad6_pos2,nad2_pos2
P27	GTR+F+I+G4	nad5_pos1,nad4L_pos1,nad4_pos1
P28	TIM+F+G4	nad4L_pos2,nad4_pos2,
P29	HKY+F+G4	nad4L_pos3
P30	TPM2u+F+G4	nad5_pos3,nad4_pos3
P31	TN+F+G4	nad6_pos3
**PCGrRNAdataset**		
Partitions	Best Model	Partition names
P1	GTR+F+G4	rrnL
P2	GTR+F+G4	rrns
P3	TIM3+F+G4	cox2_pos1
P4	TIM+F+I+G4	cox3_pos2,cytb_pos2, cox2_pos2
P5	HKY+F+I+G4	cox2_pos3
P6	GTR+F+G4	atp6_pos1,cytb_pos1, cox3_pos1
P7	HKY+F+I+G4	nad3_pos3,cox3_pos3, atp6_pos3
P8	HKY+F+G4	cytb_pos3
P9	TPM2u+F+I+G4	nad1_pos1
P10	TIM+F+I	nad1_pos2, atp6_pos2
P11	HKY+F+G4	nad1_pos3
P12	GTR+F+I+G4	atp8_pos1,nad3_pos1, nad6_pos1, nad2_pos1
P13	TVM+F+G4	nad3_pos2, nad6_pos2, nad2_pos2

P14	HKY+F+I+G4	nad2_pos3, atp8_pos3
P15	GTR+F+I+G4	nad5_pos1, nad4_pos1, nad4L_pos1
P16	TIM+F+G4	nad4L_pos2, nad4_pos2
P17	HKY+F+G4	nad4L_pos3
P18	TPM2u+F+G4	nad5_pos3, nad4_pos3
P19	GTR+F+G4	nad5_pos2
P20	TN+F+G4	nad6_pos3
P21	TPM3u+F+G4	atp8_pos2
P22	TIM2+F+G4	cox1_pos1
P23	TIM2+F+G4	cox1_pos2
P24	TPM2+F+G4	cox1_pos3

PCG dataset

Partitions	Best Model	Partition names
P1	GTR+F+G4	atp6_pos1,cytb_pos1, cox3_pos1
P2	K3Pu+F+I	atp6_pos2, nad1_pos2
P3	HKY+F+I+G4	cox2_pos3,nad3_pos3, cox3_pos3, atp6_pos3
P4	HKY+F+G4	atp8_pos1
P5	TPM3u+F+G4	atp8_pos2
P6	HKY+F+I+G4	nad2_pos3, atp8_pos3
P7	TIM2+F+I	cox1_pos1
P8	F81+F+I	cox1_pos2
P9	HKY+F+G4	cox1_pos3
P10	TIM3+F+G4	cox2_pos1
P11	TIM+F+I+G4	cox3_pos2, cox2_pos2, cytb_pos2
P12	HKY+F+G4	cytb_pos3
P13	TPM2u+F+I+G4	nad1_pos1
P14	HKY+F+G4	nad1_pos3
P15	GTR+F+I+G4	nad6_pos1, nad3_pos1, nad2_pos1
P16	TVM+F+G4	nad3_pos2, nad2_pos2, nad6_pos2
P17	GTR+F+I+G4	nad5_pos1, nad4L_pos1, nad4_pos1
P18	GTR+F+G4	nad5_pos2, nad4L_pos2, nad4_pos2
P19	HKY+F+G4	nad4L_pos3
P20	TPM2u+F+G4	nad5_pos3, nad4_pos3
P21	TN+F+G4	nad6_pos3

PCG12 dataset

Partitions	Best Model	Partition names
P1	TIM2+F+I+G4	cox3_pos1, cob_pos1, atp8_pos2, atp6_pos1
P2	F81+F+I	atp6_pos2, cox1_pos2
P3	TIM2+F+I+G4	nad2_pos1,nad3_pos1, nad6_pos1, atp8_pos1
P4	TIM2+F+I	cox1_pos1
P5	TIM3+F+G4	cox2_pos1
P6	K3Pu+F+I+G4	cob_pos2, cox2_pos2, cox3_pos2
P7	GTR+F+I+G4	nad1_pos1,nad5_pos1,nad4_pos1, ad4l_pos1
P8	GTR+F+I+G4	nad4_pos2,nad1_pos2,nad5_pos2, ad4l_pos2
P9	TVM+F+I+G4	nad6_pos2, nad2_pos2, nad3_pos2

Table S3: The length and A+T% of different components of mitogenomes in Cleridae

species	Full		PCGs		tRNA		rRNA		CR		codon position of PCG		
	length	AT%	length	AT%	length	AT%	length	AT%	length	AT%	1	2	3
Clerus sp.	>12578	78.3	11,129	78.0	>1272	81.2	>145	75.2	?	?	72.3	68.81	92.78
Clerus dealbatus	16,003	79.1	11,117	77.7	1,487	80.4	2,064	81.4	1,284	90.3	72.41	69.08	89.97
Xenorthrius discoidalis	16,153	80	11,077	78.4	1,448	80.1	2,077	82.2	1,413	89.5	72.28	68.22	93.78
Opilo grahami	16,209	80	11,118	78.4	1,447	80.1	2,076	82.1	1,470	89.2	72.4	69	93.75
Clerus klapperichi	>14691	77.7	11,104	77.1	1,458	80.4	>1778	80.1	?	?	72.42	69.11	89.46
Omadius sp.	>13417	76.1	10,182	74.4	>1,051	82.40	2,073	81.7	>161	?	69.39	71.45	81.24
Trichod sp.	16,572	78.6	11,094	76.7	1,455	80.6	2,074	81.8	1,670	85.6	71.44	69.41	89.15
Pieleus irregularis	17,127	77.7	11,059	75.1	1,442	79.6	2,086	81.4	2,401	85.4	70.3	68.64	86.04
Callimerus chinensis	16,354	78.9	11123	77.2	1,462	80.1	2070	82	1,635	86.5	71.75	68.4	91.13
Callimerus inbasalis	>15539	78.5	11,093	77.4	1,460	80.4	2,071	81.6	>126	70.8	71.92	68.8	91.26
Callimerus sp.	16,920	79.1	11,138	77.8	1,471	79.7	2,074	81.5	1,598	84.8	71.77	68.39	93.09
Neohydnus sinensis	16,533	79.5	11,128	77.4	1,451	80.9	2,080	82.8	1,700	88.2	72.19	69.03	90.61
Callimerus nigroapicalis	>19746	77.7	11,093	76.3	>1387	79.2	>352	73.9	?	?	71.7	68.39	88.42
Neohydnus jiuzhaigouensis	16,305	79.5	11,134	77.6	1,457	80.3	2,082	83.4	1,531	87.9	71.73	68.9	91.32
Tenerus flavicollis	15,638	78	11,142	76.3	1,439	79.0	2,068	81.9	987	88.3	70.75	68.19	89.77
Tenerus hilleri	15,657	77.1	11,140	75.5	1,444	78.7	2,066	81.2	1,001	84.5	69.97	68.27	88.12
Tillus nitidus	>16645	78.9	11,135	76.5	1,430	79.4	2,068	82.6	>466	86.7	71.85	68.66	88.92
Cladiscus yunnanus	16,029	78.7	11,127	77.3	1,434	79.4	2,070	81.7	1,382	85.9	71.28	68.74	91.67

Notes. ? indicates that it is uncertain due to the incompleteness.

Table S4: A+T% of each PCG of mitogenomes in Cleridae

species/genes	*atp6*	*atp8*	*cox1*	*cox2*	*cox3*	*cytb*	*nad1*	*nad2*	*nad3*	*nad4*	*nad4L*	*nad5*	*nad6*
Clerus sp.	77.8	87.2	70.1	75.5	72.6	74.3	78.8	83.3	80.8	80.2	83.4	81.6	85.1
Clerus dealbatus	77.4	83.3	69.2	76	71.2	73.6	76.8	82.7	83	80.3	79.5	80.3	84.6
Xenorthrius discoidalis	77.5	87.8	70.2	75.9	74.8	75.4	78.5	82.2	79.9	81.6	83.2	81.7	85.9
Opilo grahami	77.5	87.8	70.5	75.9	74.8	75.4	78.5	82.2	79.9	81.6	83.2	81.8	85.9
Clerus klapperichi	77	83.3	69.2	75.1	71.6	73.5	76.8	82.2	82.7	80.2	79.3	80	85.1
Omadius sp.	73.6	80.8	66.4	71.5	71.9	70.4	77.4		74.6	76.2	81.4	78.4	84.5
Trichod sp.	75.7	87.8	69.3	72.3	72.9	72.4	78	82	77.7	78.4	81.1	80.4	85.1
Pieleus irregularis	72.9	83.3	66.2	72.4	69.5	71.8	74.6	81.6	79.8	78.1	81.5	78.1	83.1
Callimerus chinensis	76.3	84.6	67.2	74.4	73	74.1	77.7	80.8	81.5	82.3	81.4	80.3	85.1
Callimerus inbasalis	75.5	87.1	67.5	75.6	72.7	74.2	78.8	81	79	81.5	82.8	80.9	86.1
Callimerus sp.	76.8	84.6	69	75.5	72.9	74.8	78.4	81.4	83	82	82.8	80.7	85.9
Neohydnus sinensis	76.2	83.9	69.4	74.6	72.7	74.6	77.7	81.3	78.7	80.9	82.5	80.4	85.9
Callimerus nigroapicalis	75.2	85.2	67.3	72.7	71.4	72.5	77.6	79.7	79.9	80.7	80.7	79.7	83.7
Neohydnus jiuzhaigouensis	76.8	84.6	69.4	73.2	73.1	74.5	78.5	80.8	80.7	81.1	81.7	81.4	85.5
Tenerus flavicollis	75	85.2	67.3	70.5	72.7	72.5	77.6	79.5	78.7	80.8	82.4	80.8	83.3
Tenerus hilleri	75.6	82.7	66.5	73.4	71.8	71.1	76.5	77.8	76.9	80.1	82.5	79.9	80.1
Tillus nitidus	75.4	84.6	69.1	74.6	71.1	72.9	77.4	80.4	77.4	80.6	79.3	79.8	84.3
Cladiscus yunnanus	77	87.2	68.8	73.5	73.5	74.3	77.4	80.6	77.7	80.9	83.5	80.9	85.5

Table S5: A+T% and A+T skew, C+G% and G+C skew in the 13 PCGs of mitogenomens in Cleridae.

species/content	A+T%	A+T skew	C+G%	G+C skew
Clerus sp.	78	0.017949	22	-0.13636
Clerus dealbatus	77.2	0.023316	22.8	-0.15789
Xenorthrius discoidalis	78.4	-0.0051	21.6	-0.12963
Opilo grahami	78.4	-0.0051	21.6	-0.12963
Clerus klapperichi	77.1	0.022049	22.9	-0.15284
Omadius sp.	74.3	0.033647	25.7	-0.18677
Trichodes sp.	76.7	0.014342	23.3	-0.19313
Pieleus irregularis	75.1	-0.00133	24.9	-0.18072
Callimerus chinensis	76.9	-0.01691	23.2	-0.14655
Callimerus inbasalis	77.4	0.005168	22.6	-0.16814
Callimerus sp.	77.8	-0.00514	22.2	-0.14414
Neohydnus sinensis	77.4	-0.00775	22.6	-0.15044
Callimerus nigroapicalis	76.3	0.006553	23.8	-0.16807
Neohydnus jiuzhaigouensis	77.6	-0.00773	22.5	-0.13778
Tenerus flavicollis	76.3	0.030144	23.7	-0.18987
Tenerus hilleri	75.5	0.035762	24.5	-0.22449
Tillus nitidus	76.6	0.002611	23.5	-0.18298
Cladiscus yunnanus	77.3	0.001294	22.8	-0.16667

Table S6: Codon usage of PCGs of mitogenomes in Cleridae.

	Start codon						Stop codon		
genes	ATT	ATA	ATG	ATC	TTG	GTG	TAG	TAA	T-
nad2	14	3					2	15	
cox1	10	2	5					17	1
cox2	6	6	3	2		1		9	9
atp8	13			5				18	
atp6		18						18	
cox3			18					9	9
nad3	7	11					6	12	
nad5	5	13					1	3	14
nad4	2	15	1						18
nad4L	3		14		1		2	16	
nad6	14	2		2				18	
cytb			18				11	5	2
nad1	2				16		17	1	

Table S7: RSCU of the mitochondrial PCGs of Cleridae

	Clerinae		Hydnocerinae		Korynetinae		Tillinae		Average
AA	Codon	RSCU	Codon	RSCU	Codon	RSCU	Codon	RSCU	RSCU
Phe	UUU	1.81125	UUU	1.84	UUU	1.79	UUU	1.76	1.800313
Phe	UUC	0.18875	UUC	0.16	UUC	0.21	UUC	0.24	0.199688
Leu2	UUA	4.76375	UUA	4.92	UUA	4.34	UUA	4.6	4.655938
Leu2	UUG	0.3525	UUG	0.25	UUG	0.27	UUG	0.28	0.288125
Leu1	CUU	0.43	CUU	0.46	CUU	0.705	CUU	0.65	0.56125
Leu1	CUC	0.04125	CUC	0.04	CUC	0.125	CUC	0.07	0.069063
Leu1	CUA	0.375	CUA	0.31	CUA	0.52	CUA	0.385	0.3975
Leu1	CUG	0.03125	CUG	0.02	CUG	0.035	CUG	0.02	0.026563
Ile	AUU	1.85625	AUU	1.85	AUU	1.81	AUU	1.86	1.844063
Ile	AUC	0.14375	AUC	0.16	AUC	0.19	AUC	0.14	0.158438
Met	AUA	1.82375	AUA	1.84	AUA	1.855	AUA	1.855	1.843438
Met	AUG	0.17625	AUG	0.16	AUG	0.145	AUG	0.145	0.156563
Val	GUU	1.9675	GUU	1.93	GUU	2.04	GUU	1.83	1.941875
Val	GUC	0.17875	GUC	0.15	GUC	0.265	GUC	0.13	0.180938
Val	GUA	1.685	GUA	1.77	GUA	1.52	GUA	1.885	1.715
Val	GUG	0.165	GUG	0.15	GUG	0.18	GUG	0.165	0.165
Ser2	UCU	3.07875	UCU	2.92	UCU	2.66	UCU	2.68	2.834688
Ser2	UCC	0.2875	UCC	0.27	UCC	0.365	UCC	0.395	0.329375
Ser2	UCA	1.80125	UCA	2.1	UCA	2.165	UCA	1.99	2.014063
Ser2	UCG	0.08875	UCG	0.1	UCG	0.09	UCG	0.11	0.097188
Pro	CCU	2.52875	CCU	2.34	CCU	2.07	CCU	2.475	2.353438
Pro	CCC	0.34625	CCC	0.4	CCC	0.55	CCC	0.335	0.407813
Pro	CCA	1.025	CCA	1.17	CCA	1.26	CCA	1.11	1.14125
Pro	CCG	0.10125	CCG	0.1	CCG	0.12	CCG	0.08	0.100313
Thr	ACU	1.91	ACU	2.16	ACU	2.075	ACU	2.24	2.09625
Thr	ACC	0.43875	ACC	0.3	ACC	0.34	ACC	0.31	0.347188
Thr	ACA	1.55625	ACA	1.5	ACA	1.56	ACA	1.4	1.504063
Thr	ACG	0.095	ACG	0.04	ACG	0.025	ACG	0.05	0.0525
Ala	GCU	2.18	GCU	2.15	GCU	2.52	GCU	2.275	2.28125
Ala	GCC	0.47875	GCC	0.41	GCC	0.41	GCC	0.435	0.433438
Ala	GCA	1.20375	GCA	1.37	GCA	1.055	GCA	1.245	1.218438
Ala	GCG	0.13875	GCG	0.08	GCG	0.015	GCG	0.045	0.920938
Tyr	UAU	1.80875	UAU	1.79	UAU	1.75	UAU	1.74	1.772188
Tyr	UAC	0.19125	UAC	0.21	UAC	0.25	UAC	0.26	0.227813
His	CAU	1.75875	CAU	1.74	CAU	1.5	CAU	1.77	1.692188
His	CAC	0.24125	CAC	0.27	CAC	0.5	CAC	0.23	0.310313
Gln	CAA	1.83625	CAA	1.88	CAA	1.795	CAA	1.81	1.830313
Gln	CAG	0.16375	CAG	0.12	CAG	0.205	CAG	0.19	0.169688
Asn	AAU	1.77375	AAU	1.79	AAU	1.725	AAU	1.75	1.759688
Asn	AAC	0.22625	AAC	0.21	AAC	0.275	AAC	0.25	0.240313
Lys	AAA	1.7375	AAA	1.79	AAA	1.785	AAA	1.855	1.791875
Lys	AAG	0.2625	AAG	0.21	AAG	0.215	AAG	0.145	0.208125
Asp	GAU	1.62375	GAU	1.68	GAU	1.71	GAU	1.72	1.683438
Asp	GAC	0.37625	GAC	0.32	GAC	0.29	GAC	0.28	0.316563
Glu	GAA	1.73375	GAA	1.83	GAA	1.705	GAA	1.87	1.784688
Glu	GAG	0.26625	GAG	0.18	GAG	0.295	GAG	0.13	0.217813

Cys	UGU	1.6925	UGU	1.81	UGU	1.825	UGU	1.79	1.779375
Cys	UGC	0.3075	UGC	0.19	UGC	0.175	UGC	0.21	0.220625
Trp	UGA	1.82875	UGA	1.88	UGA	1.805	UGA	1.82	1.833438
Trp	UGG	0.17125	UGG	0.12	UGG	0.195	UGG	0.18	0.166563
Arg	CGU	1.37	CGU	1.32	CGU	1.305	CGU	1.425	1.355
Arg	CGC	0.17	CGC	0.11	CGC	0.23	CGC	0.19	0.175
Arg	CGA	2.2175	CGA	2.26	CGA	2.305	CGA	2.035	2.204375
Arg	CGG	0.24	CGG	0.31	CGG	0.15	CGG	0.345	0.26125
Ser1	AGU	0.51875	AGU	0.53	AGU	0.56	AGU	0.61	0.554688
Ser1	AGC	0.10125	AGC	0.07	AGC	0.08	AGC	0.045	0.074063
Ser1	AGA	2.0225	AGA	1.89	AGA	1.93	AGA	2.02	1.965625
Ser1	AGG	0.09875	AGG	0.11	AGG	0.15	AGG	0.145	0.125938
Gly	GGU	0.86	GGU	1.02	GGU	0.955	GGU	0.8	0.90875
Gly	GGC	0.1875	GGC	0.16	GGC	0.14	GGC	0.15	0.159375
Gly	GGA	2.05625	GGA	2.12	GGA	2.115	GGA	2.345	2.159063
Gly	GGG	0.8925	GGG	0.7	GGG	0.79	GGG	0.7	0.770625

Table S8: Amino acid constituents among four subfamilies of Cleridae

AA	Clerinae	Hydnocerinae	Korynetinae	Tillinae	Average
Leu2(L2)	489	499	437	475	475
Ile(I)	398	402	417	409	406
Phe(F)	355	374	363	360	363
Met(M)	254	257	248	263	256
Ser2(S2)	214	233	231	233	228
Asn(N)	207	209	210	206	208
Gly(G)	187	194	203	197	195
Tyr(Y)	186	175	171	176	177
Thr(T)	154	156	169	159	159
Val(V)	159	156	167	157	160
Ala(A)	147	152	142	138	145
Ser1(S1)	112	113	119	127	117
Pro(P)	130	132	132	126	130
Lys(K)	115	114	112	115	114
Leu1(L1)	85	80	131	110	101
Trp(W)	93	94	97	96	95
Glu(E)	77	76	74	77	76
His(H)	68	71	68	69	69
Asp(D)	64	64	66	65	64
Gln(Q)	64	61	64	63	63
Arg(R)	51	52	52	52	52
Cys(C)	31	33	34	31	32
